# Design and Experimental Research of a Rotary Micro-Actuator Based on a Shearing Piezoelectric Stack

**DOI:** 10.3390/mi10020096

**Published:** 2019-01-29

**Authors:** Hehe Huang, Longfei Wang, Ying Wu

**Affiliations:** State Key Laboratory for Strength and Vibration of Mechanical Structures, National Demonstration Center for Experimental Mechanics Education, Shaanxi Engineering Laboratory for Vibration Control of Aerospace Structures, School of Aerospace Engineering, Xi’an Jiaotong University, Xi’an 710049, China; huanghehe@stu.xjtu.edu.cn (H.H.); wlfpal@stu.xjtu.edu.cn (L.W.)

**Keywords:** micro-actuator, shearing piezoelectric stack, inertia

## Abstract

The working principle of a rotating micro-actuator based on a piezoelectric stack was theoretically analyzed and experimentally verified. The actuator is compact in structure, and the key component is the shearing piezoelectric stack. The piezoelectric stack is used to drive the micro-rotor via an electromechanical transition, which produces high-speed rotation of the micro-rotor. We first established the dynamic model of the micro-actuator and numerically analyzed the motion of this model. The step displacement output was observed by simulation, and the step increment is quite large. For experimental verification, we fabricated the piezoelectric micro-actuator with a size of 12 mm × 10 mm × 8 mm and mass of 4.12 g and conducted a series of experiments. The results show qualitative agreement with the theoretical results; the maximum output speed of the micro-actuator is 5.86 × 10${}^{5}$ μrad/s, and the motion resolution is 0.64 μrad, which is greater than that of most traditional piezoelectric actuators. The proposed micro-actuator offers superior performance in driving of selected small objects, such as in micro-/nano-processing and cell operation.

## 1. Introduction

Piezoelectricity is the electric charge that accumulates in certain solid materials in response to an applied mechanical stress. The piezoelectric effect of such materials highlights the process of transformation from mechanics to electronics and the reversibility of the process. In other words, piezoelectric materials can deform based on the effect of the electric field and can even produce high-frequency periodic deformation under alternating current. The properties of piezoelectric materials are used in certain piezoelectric precision actuators [1,2]. Such actuators have the advantages of quick response, high resolution, and resistance to electromagnetic interference, which attracts their use in selected fields of precise optical alignment, microbiological cell operation [3,4], ultra-precision machining [5,6], micro-/nano-mechanical testing [7,8], and atomic force microscopy (AFM) [9,10], among others.

Based on different driving modes, piezoelectric precision actuators can be divided into four categories: ultrasonic, direct drive, inchworm and inertial types [11,12,13,14,15,16,17,18]. Among these, the ultrasonic piezoelectric actuator [19] often requires a high driving voltage. The direct driving piezoelectric actuator [20,21] can only produce tens of microns of displacement. The control system of an inchworm-type piezoelectric actuator [22,23,24,25,26,27] is highly complex due to coordination of multiple structures, and it cannot be driven by high-frequency alternating voltage. In contrast, the inertial piezoelectric actuator [28,29,30] offers the advantages of high precision, large stroke, high working frequency and rapid motion speed and is also conveniently driven. Therefore, many researchers have performed studies on inertial piezoelectric actuators.

Hii [30] developed an inertial piezoelectric actuator based on the principle of stick–slip. The entire structure consists of six shear piezoelectric blocks that can produce high-precision linear displacement, but the working frequency is low, and the maximum speed reaches only 13.5 μm/s. Gao [18] developed an inertial piezoelectric actuator that could produce both axial displacement and angular displacement. Cheng [31] used an asymmetric clamping structure to develop an inertial rotation piezoelectric actuator with high precision and stability. However, with the development of technologies such as MEMS systems and micro/nano-processing, an increasing demand has arisen for large-stroke and high-precision micro-actuators. Current piezoelectric actuators are often difficult to use in these fields due to their complicated structure and large size.

In the field of micro-piezoelectric actuators, The Physikinstrumente (PI) company’s Q-motion piezoelectric actuator series (Physik Instrumente (PI) GmbH and Co, Karlsruhe, Germany) offers relatively outstanding performance, such as large output force and small size, but cannot maintain the small size while ensuring high resolution. For example, Q-614.900 is small in size (18 mm × 18 mm × 10 mm), but it has a low resolution (1 μrad). The resolution of Q-632.930 can reach 0.75 μrad, but the overall size is too large (32 mm × 32 mm × 7 mm). The M3-RS rotary table from NewScale Technologies (New Scale Technologies, Inc., Victor, NY, USA) is small in size, but the resolution is only 100 μrad. Therefore, it is difficult for a piezoelectric actuator to maintain high precision while ensuring small size, and these features restrict their application. To overcome these problems, in this study, we propose a micro-inertial piezoelectric actuator based on the principle of stick–slip. This actuator offers high resolution and a large stroke, produces a higher output rotation speed, and has a more compact structure. The overall size of the actuator is less than 12 mm, the height is only 8 mm, and the motion resolution is 0.64 μrad, which is higher than that of most traditional piezoelectric actuators. The remainder of this paper is organized as follows. Section 2 of the article introduces the structural design of the piezoelectric micro-actuator. Section 3 establishes the dynamic model of the micro-actuator and investigates the dynamic properties using numerical methods. Experimental verification is illustrated in Section 4. Finally, Section 5 presents conclusions.

## 2. Structure and Operational Principle

**Figure 1:**
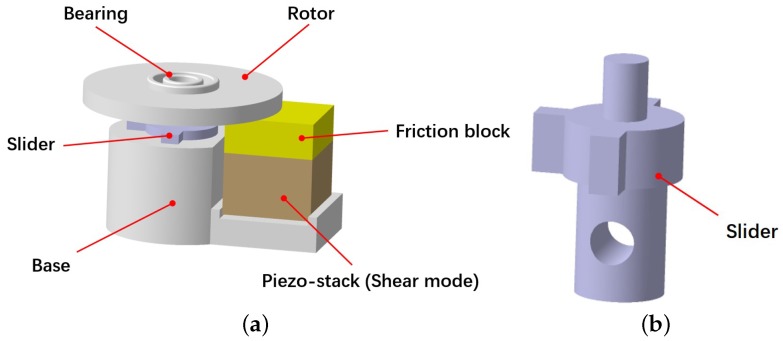
Structural diagram of the piezoelectric actuator: (**a**) overall structure; and (**b**) slider.

**Figure 2:**
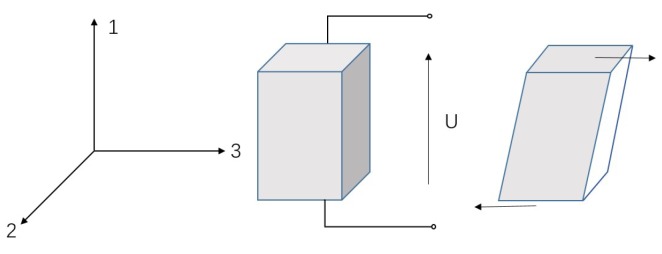
Schematic diagram of the PZT (shear mode).

The structural diagram of the proposed piezoelectric micro-actuator is shown in Figure 1. The device is primarily composed of a bearing, a rotor, a slider, a base, a friction block and a PZT(shear mode). The upper end of the slider is connected with the bearing, and the rotor is connected with the bearing through an interference fit. The slider can slide freely along the vertical slots of the base without rotating because they are nested with each other. The lower portion of the slider contains a circular hole such that it can also be used to adjust the preload between the friction block and the rotor via hanging of heavy objects or adding magnets. The PZT stack is fixed on the base, the top of which is pasted with a friction block. The PZT stack can undergo shearing deformation under an applied electric field (see Figure 2). With the effects of the weight of the bearing, rotor and slider, the rotor and the friction block can remain in good contact. Therefore, the rotor begins to rotate when the PZT is deformed under the electric field.

**Figure 3:**
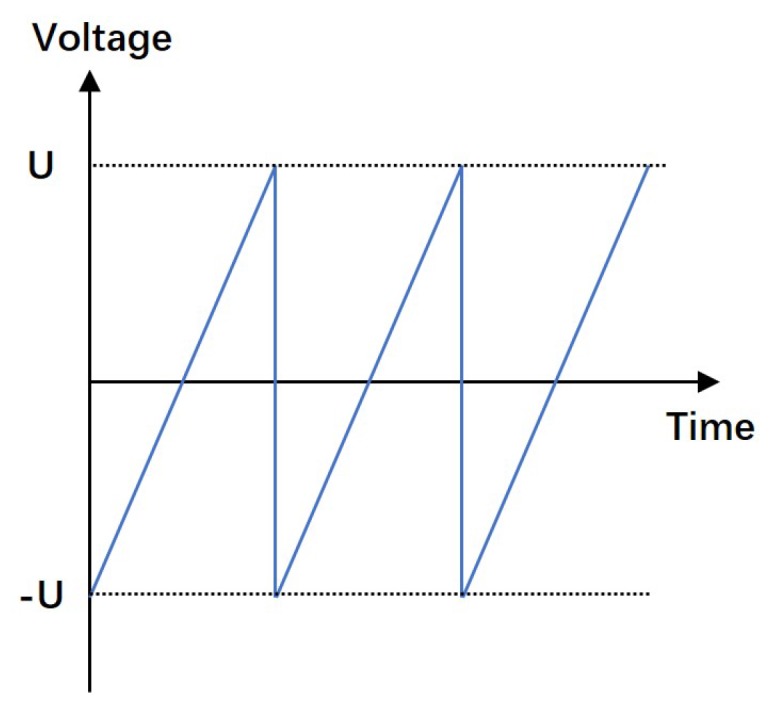
Input voltage of PZT.

**Figure 4:**
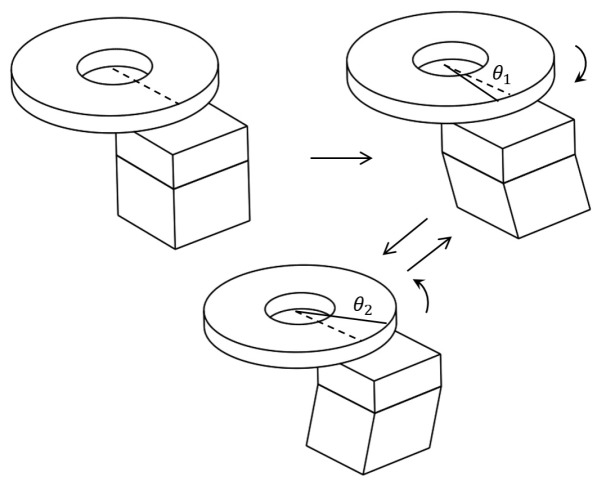
Working principle of the proposed micro-actuator.

In this paper, a serrated type of external electric field is applied to drive the shear deformation of the PZT stack, as shown in Figure 3. The working process is shown in Figure 4, and it can be divided into three steps as follows:

(1) The serrated-type voltage is not applied to the PZT stack at the beginning, and thus the PZT stack maintains the cubic shape, and the rotor remains in a static state.
(2) As the serrated-type voltage is applied to the PZT stack, the voltage rapidly changes from 0 to −U (see Figure 3), and the PZT stack produces shear deformation, which leads to movement of the friction block via the dynamic friction between them. The rotor can only rotate through a small angle of $\theta_{1}$ along the counterclockwise direction because the response time of this step is quite short.
(3) The serrated-type voltage on the PZT subsequently increases slowly from −U to U, and in this manner, the PZT stack can slowly deform along the clockwise tangential direction of the rotor. With the effects of friction between the rotor and the friction block, the rotor can rotate in the opposite direction with a rotation angle of $\theta_{2}$. Thus, the total angular displacement θ of each cycle is written as follows:
  (1) $$\theta =\theta_{2}-\theta_{1}$$

By repeating Steps 2 and 3, the actuator can continue to rotate at a certain frequency for a long period of time. In addition, the inverse rotation output of the rotor can be obtained by inputting the reverse serrated-type voltage.

## 3. Analysis

**Figure 5:**
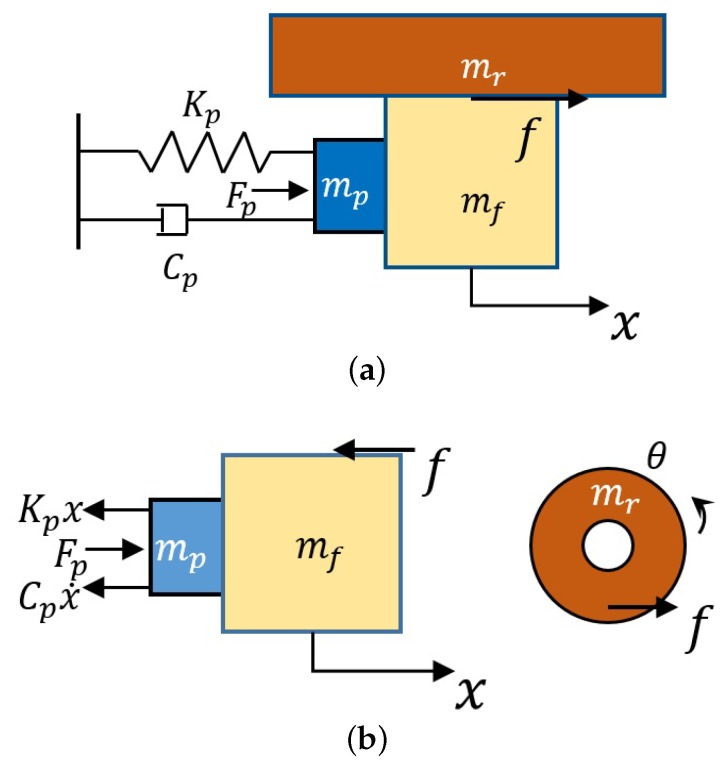
(**a**) Overall dynamic model of the mechanism; and (**b**) force analysis of the friction body and the PZT stack and force condition of the rotor.

In this paper, a mathematical model is established to further analyze the displacement output of the actuator, and the model is shown in Figure 5.

In this model, $m_{p}$ represents the equivalent mass of PZT stack, $m_{f}$ represents the mass of the friction body, $m_{r}$ is the mass of rotor, $C_{p}$ is the damping coefficient of the PZT stack, $F_{p}$ is the force supplied by the PZT stack under the electric field, and $K_{p}$ is the equivalent stiffness of the PZT stack and is related to the shear modulus *G*, cross-sectional area *A* and height *h* of the PZT stack, which can be expressed by the following formula:

(2) $$K_{p}=\xi \frac{GA}{h}$$

where ξ is the coefficient of the error corrections caused by the uneven contact surface and shear force distribution. According to Figure 5, the dynamic model of the proposed micro-actuator can be written as follows:

(3) $$\left(m_{p}+m_{f}\right)\ddot{x}+C_{p}\dot{x}+K_{p}x=F_{p}\left(t\right)-f\left(t\right)$$

(4) $$J\ddot{\theta }+C_{r}\dot{\theta }+K_{r}\theta =f\left(t\right)R$$

where *J* is the moment of inertia of the rotor, *R* is the distance between the equivalent point of force and the center of the rotor, f(t) is the equivalent friction force, $C_{r}$ is the damping of the rotor, and $K_{r}$ is the equivalent stiffness of the rotor.

The equivalent friction force f(t) of the stick–slip piezoelectric micro-actuator is quite difficult to describe because many factors should be considered, including the contacting surface condition, relative sliding velocity, friction lag, etc. Instead of the conventional Coulomb friction theorem, we use the LuGre friction model [32] in our study because it contains most of the frictional features observed in the experiment. By substituting $\left(R\dot{\theta }-\dot{x}\right)$ with ν, the friction force f(t) can be expressed as shown:

(5) $$f\left(t\right)=\sigma_{0}z+\sigma_{1}\dot{z}+\sigma_{2}\nu$$

where $\sigma_{0},\sigma_{1},\sigma_{2}$ are the stiffness of the bristles, the damping coefficient and the viscous friction coefficient, respectively; *v* represents the relative speed of the rotor and the friction block at the equivalent contact point; and *z* represents the average deflection of the bristles and can be written in the following form:

(6) $$\dot{z}=\nu -\frac{\left|\nu \right|}{g(\nu )}\sigma_{0}z$$

(7) $$g\left(v\right)=F_{c}-\left(F_{s}-F_{c}\right)e^{-\frac{\nu }{\nu_{s}}}$$

where $F_{c}$ is the Coulomb friction force, $F_{s}$ is the maximum static friction, function g(ν) represents the Stribeck effect, and $\nu_{s}$ is the Stribeck velocity. For PZT, the output force under electric field can be expressed as follows:

(8) $$\Delta L=nd_{15}V$$

(9) $$F_{p}=\Delta LK_{p}$$

where $d_{15}$ is the piezoelectric constant of the shearing mode, *V* is the voltage applied to PZT, *n* is the number of piezoelectric stacks, and ΔL is the stroke of the PZT.

Due to the hysteresis nonlinearity of the piezoelectric material, $d_{15}$ is not a fixed value in the shear mode, and it is related to the voltage variation [30,33,34]. The variation characteristics can be described by Equation (10)

(10) $$d_{15}=d_{15}^{0}+\alpha V$$

where $d_{15}^{0}$ is the piezoelectric constant at low voltage, and α is a constant related to the piezoelectric constant and voltage change.

**Figure 6:**
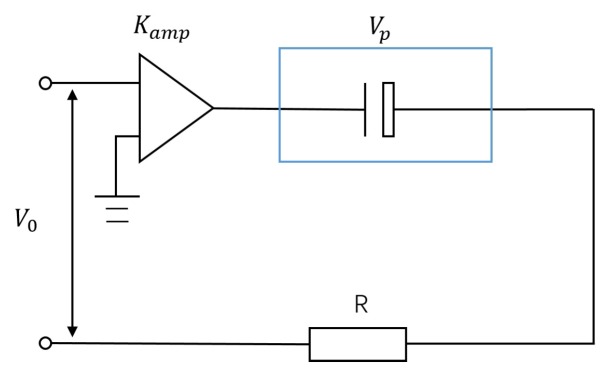
Drive circuit of the PZT stack.

However, the influence of alternating electric field should be considered, and the actual simplified driving circuit of PZT is shown in Figure 6. According to the principle of electrotechnology, the relationship between the input voltage of the PZT stack and the input voltage of the signal generator can be obtained:

(11) $$V_{p}=K_{amp}V_{0}\left(1-e^{-\frac{t}{RC}}\right)$$

where $K_{amp}$ represents the magnification of the voltage amplifier, and $V_{0}$ and $V_{p}$ are the input voltages of the signal generator and the PZT, respectively.

Equations (3) and (4) is the model of the rotary micro-actuator, and the numerical solution of the dynamic model can be obtained via determination of the key parameters. These key parameters are partially determined through experiments, such as $d_{15}^{0}$, α and certain parameters relating to the manufacturer’s specifications of the shear piezoelectric stack. Therefore, we should obtain these parameters using experiments in the following study.

## 4. Experiments

A series of experiments were conducted to evaluate the characteristics of the proposed piezoelectric micro-actuator. We first introduce our experimental platform and test the characteristics of the shear piezoelectric stack for selected key parameters. We explore the stepping characteristics of the micro-actuator by varying the conditions of the driving frequency, driving voltage, and preload.

### 4.1. Experimental System

**Figure 7:**
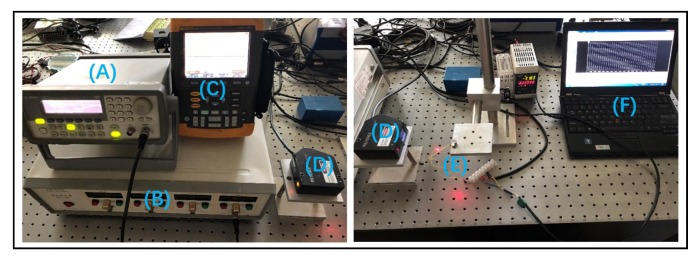
Experimental system: (**A**) signal generator; (**B**) amplifier; (**C**) oscilloscope; (**D**) laser sensor; and (**E**) actuator prototype, (**F**) PC.

**Figure 8:**
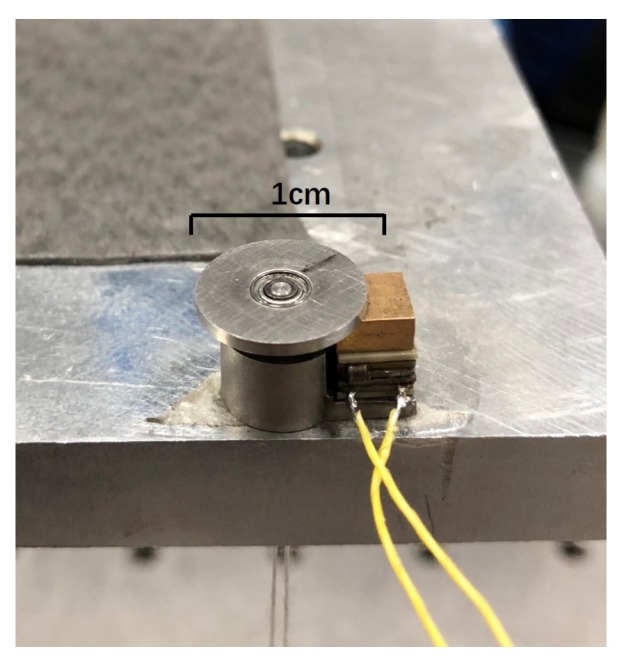
Prototype of the micro-piezoelectric actuator.

**Figure 9:**
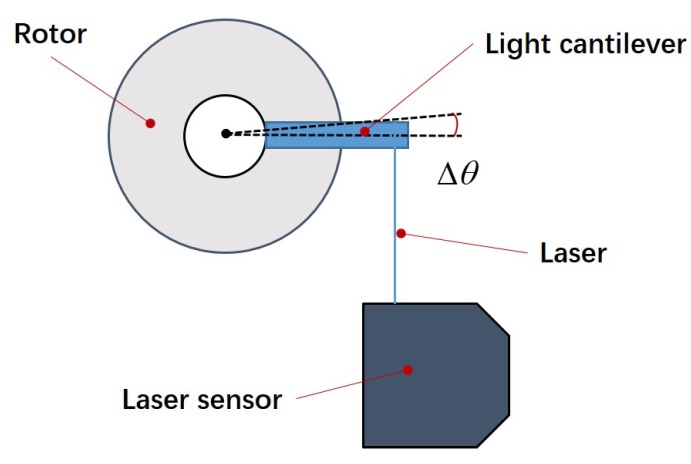
Principle of angular displacement measurement.

The experimental platform is shown in Figure 7 and consists of a signal generator, a voltage power amplifier, a laser sensor (LK-G80, Keyence Company, Osaka, Japan), a scopemeter (Fluke 196C, Fluke Company, Everett, WA, USA), a prototype, and a PC used to display the data. The serrated-type voltage signal is generated by the signal generator, and the voltage is amplified to an appropriate value for actuator driving. The prototype of the piezoelectric micro-actuator is shown in Figure 8. The overall size is 12 mm × 10 mm × 8 mm, and the mass is 4.12 g. Because the laser sensor can only be used to measure the linear displacement, a lightweight plastic cantilever beam is attached to the rotor to measure the rotation angle of the actuator (see Figure 9). At the beginning, the plastic cantilever is perpendicular to the laser. By measuring the displacement of cantilever beam in a small angle range near a vertical position (because the deflection angle is rather small, the displacement of the laser point on the light cantilever is approximately straight), the angle of the rotor can be calculated.

### 4.2. Characteristics of the Shear Piezo-Stack

**Figure 10:**
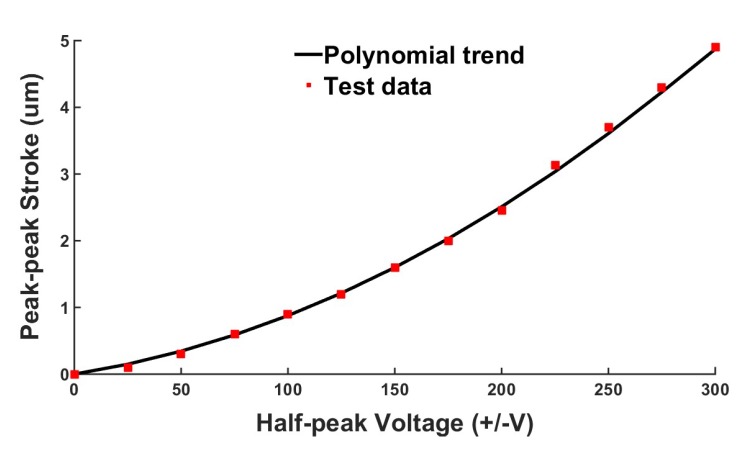
Free displacement of the shear piezoelectric stack from −V to +V voltage.

Due to the hysteresis of the piezoelectric materials, the stroke of the PZT and the applied voltage are approximately linear at low voltage, but when the voltage is high, the results are obviously nonlinear. For verification, the relationship between the stroke of the shear piezoelectric stack and the applied voltage without preload is shown in Figure 10. The peak–peak stroke refers to the shear displacement of the top of piezoelectric stack due to the effects of the voltage change from −V to +V. For convenience of description, we use the voltage U to represents the voltage change from −V to +V. Under a voltage of 300 V, the shear piezoelectric stack reaches the maximum stroke of 4.9 μm. According to Equations (8)–(10) and the experimental data of Figure 10, we found that the values of $d_{15}^{0}$ and α are 625 × 10${}^{-6}$ μm/V and 4.715 × 10${}^{-6}$ μm/V${}^{2}$, respectively. The piezoelectric constant $d_{15}^{0}$ under low voltage shows only a small difference from the manufacturer’s specifications of 550 × 10${}^{-6}$ μm/V. The free shear displacement of piezoelectric stack can be obtained as follows:

(12) $$\Delta L=3.772\times 10^{-5}\;V^{2}+0.005\;V$$

**Figure 11:**
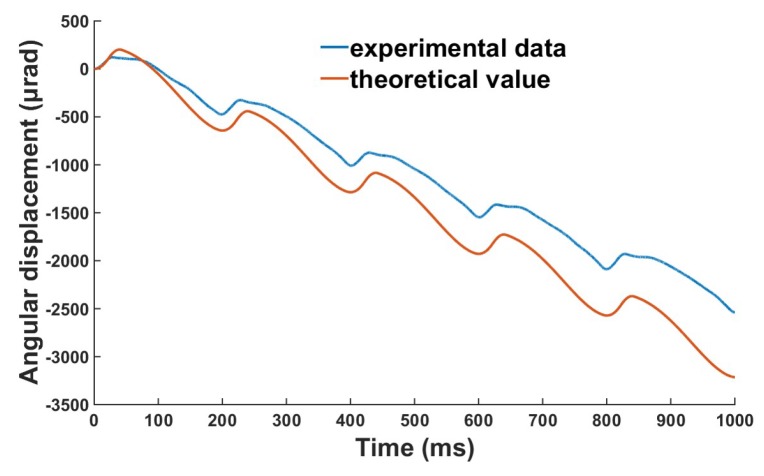
Comparison of theoretical results and experimental results at a voltage of 300 V and frequency of 5 Hz.

The theoretical results of step displacement of the piezoelectric micro-actuator can be obtained from Equations (3), (4) and (12), and the comparison between the theoretical and experimental results at the given voltage of 300 V with a frequency of 5 Hz is shown in Figure 11. Figure 11 shows that the experimental results are in good agreement with the theoretical results, but the theoretical value is slightly larger than the experimental value, mainly because the friction torque of bearing is not considered in the numerical simulation.

### 4.3. Stepping Rotation Characteristics

#### 4.3.1. Driving Frequency

**Figure 12:**
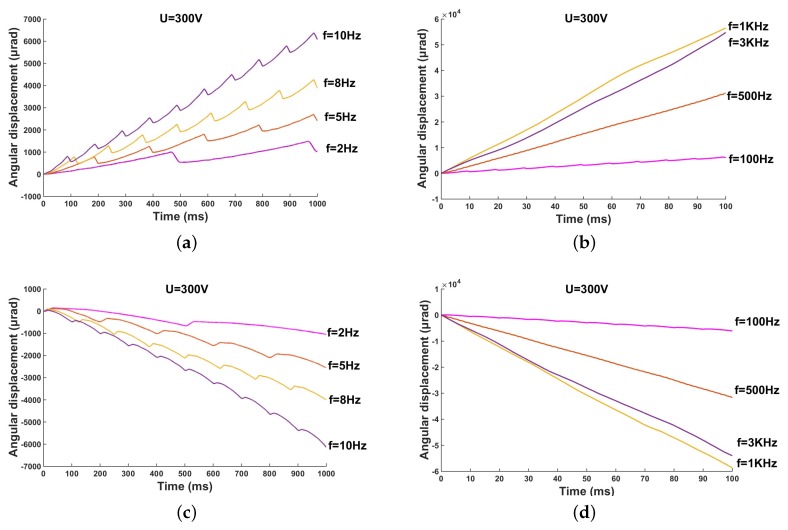
Angular displacement under various driving frequencies: (**a**) low frequency, clockwise rotation; (**b**) high frequency, clockwise rotation; (**c**) low frequency, counterclockwise rotation; and (**d**) high frequency, counterclockwise rotation.

The stepping rotation displacement of the proposed micro-actuator under different driving frequencies with a fixed voltage of 300 V and preload of 0.11 N is shown in Figure 12. Figure 12a,b indicates the clockwise stepping angle of the rotor, and the counterclockwise stepping angles of the rotor under the driving voltage are shown in Figure 12c,d. Because the shear PZT stack has a higher resonant frequency and faster response speed compared with traditional piezoelectric ceramics, it can be driven at a higher frequency.

In the experiment, the minimum driving frequency is 2 Hz, and the maximum driving frequency is 3 kHz. It can be observed that the driving frequency has a significant effect on the step angle. At low frequency, the rotor shows a more obvious retreat. As the frequency increases, as shown in Figure 12b,d, good linear step angle results can be noted in the time response. In the range from 2 Hz to 1 kHz, the step angle obviously increases together with the increment of the driving frequencies. However, when the frequency reaches 3 kHz, the step angle no longer increases, and it is slightly lower than that under a frequency of 1 kHz. This result occurs because the frequency of 3 kHz is too high, and thus the process of charging and discharging of the PZT stack is incomplete. As a result, the best driving effect cannot be achieved. Moreover, the effective driving frequency of 1 kHz is still higher than that of most of the current piezoelectric actuators, especially in the case of small size. Therefore, the micro-actuator has a faster stepping angle output, and the actuator can reach a 58,623 μrad step out within 100 ms at a frequency of 1 kHz.

#### 4.3.2. Driving Voltage

**Figure 13:**
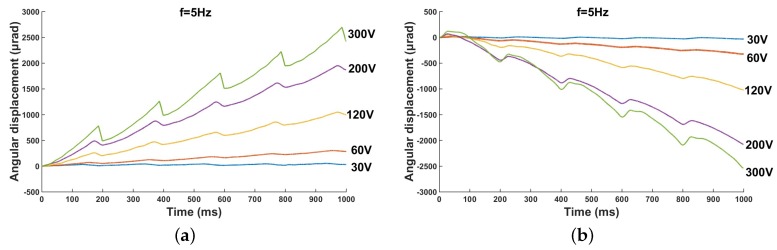
Angular displacement under various driving voltages: (**a**) clockwise rotation; and (**b**) counterclockwise rotation.

The performance of the micro-actuator is affected not only by the driving frequency but also by the amplitude of the driving voltage. The characteristics of the micro-actuator under different voltages with a driving frequency of 5 Hz and a preload of 0.11 N are shown in Figure 13. The amplitude of the driving voltage changes from 30 V to 300 V. With the increase in voltage amplitude, the angular displacement increases from 6.01 μrad to 547.8 μrad. Because the value of the preload has a great influence on the driving performance of the prototype, 6.01 μrad is not the minimum step size of the driver.

#### 4.3.3. Influence of Preload

**Figure 14:**
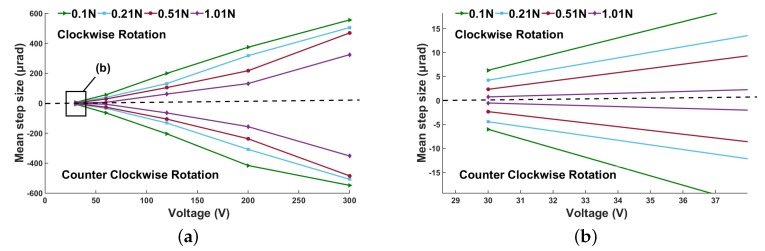
Mean step size depending on voltage for different preloads: (**a**) mean step size versus voltage for different preloads; and (**b**) magnified data for low voltage.

**Figure 15:**
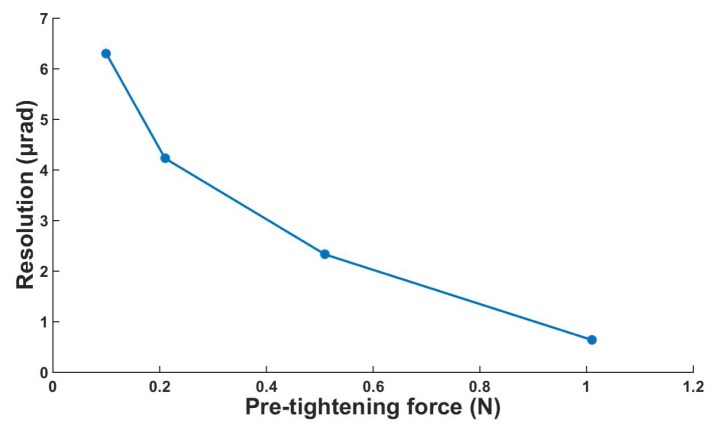
The relationship between resolution and pre-tightening force.

**Figure 16:**
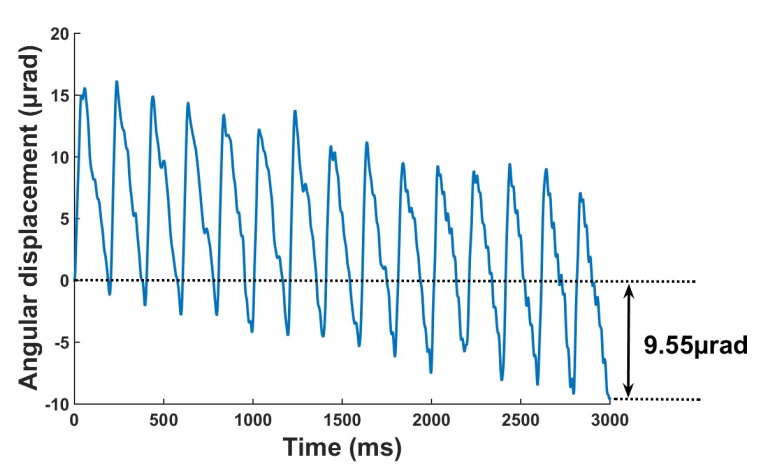
Angular displacement under a driving voltage of 30 V with a preload of 1.01 N and a driving frequency of 5 Hz.

To study the stepping performance of the actuator in the experiment, we can adjust the pretightening force via the hanging mass block. The influence of the preload on the mean step size of the actuator is shown in Figure 14a. It can be observed in Figure 14a that the mean step size decreases with increasing preload. When the voltage is 300 V and the preload is 0.11 N, the mean step size reaches the maximum value of 547.8 μrad. The local amplified data of Figure 14a are shown in Figure 14b. The results show that the minimum mean step size is 0.64 μrad with the given voltage of 30 V and a pretightening force of 1.01 N. Figure 15 shows the relationship between resolution and preload. As can be seen from the figure, the resolution increases with the increase of the preload. It should be noted that when the pre-tightening force exceeds 1.01 N, the driving effect of 30 V voltage is difficult to realize because of the large friction. The actuator’s resolution can be easily adjusted by changing the preload. However, the output of the actuator will be limited by too much pre-tightening force. Therefore, we can adjust the preload within limits to make it work in different situations. The specific driving performance under the voltage of 30 V and pre-tightening force of 1.01 N is shown in Figure 16. A relatively stable angular displacement of 9.55 μrad is obtained in 15 cycles. When the voltage is less than 30 V, the prototype cannot produce a stable output because of the small driving force, and thus the motion resolution of the actuator is 0.64 μrad. By adjusting the driving frequency, voltage, and preload, the output of the micro-actuator can be controlled.

#### 4.3.4. Rotation Speed

**Figure 17:**
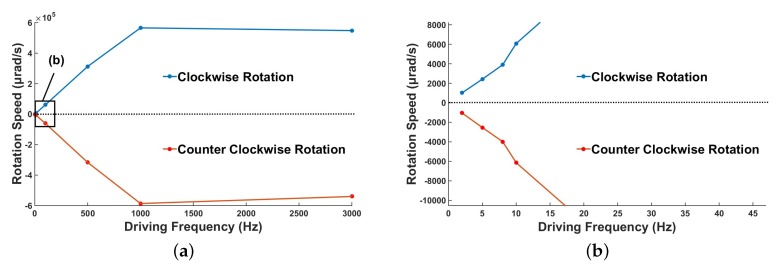
Rotation speed under various driving frequencies: (**a**) rotation speed for a driving frequency of 2 Hz to 3 KHz; and (**b**) magnified data for low voltage.

Because the driving speed is also important to the piezoelectric micro-actuators, the relationship between the rotation speed and the driving frequency of the prototype under a preload of 0.11 N and a voltage of 300 V is shown in Figure 17a. When the driving frequency is 1 kHz, the rotation speed is the largest, and the maximum value is 5.86 × 10 ${}^{5}$ μrad/s. If the driving frequency is increased to 3 KHz, the shear PZT stack cannot charge and discharge completely, and thus the rotation speed is slightly lower than that at a frequency of 1 kHz. Figure 17b presents a partial enlargement map of Figure 17a, which shows the relationship between the rotation speed and the low driving frequency. When the driving frequency increases from 2 Hz to 10 Hz, the rotation speed increases from 1048.11 μrad/s to 6141 μrad/s. It can be concluded that the actuator has a notably wide rotation speed output span.

#### 4.3.5. Maximum Output Torque

**Figure 18:**
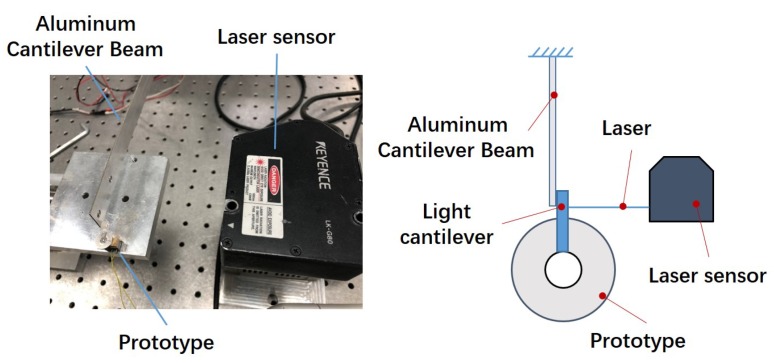
Measuring principle of output torque.

**Figure 19:**
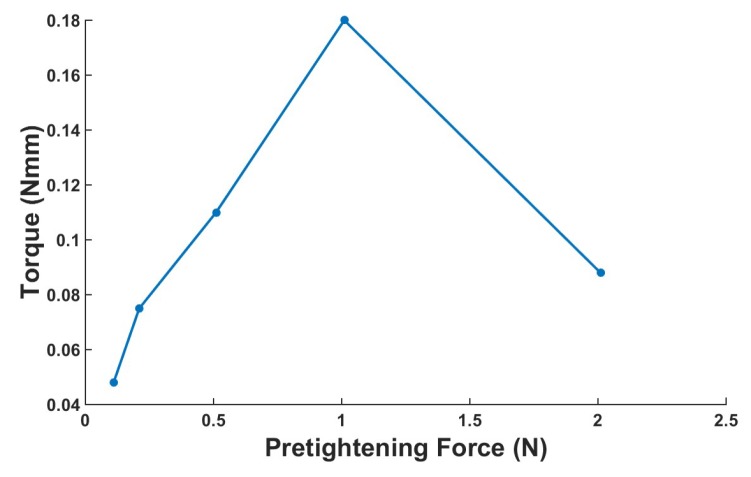
Maximum output torque under different pretightening forces.

To obtain the maximum output torque of the actuator, the test platform shown in Figure 18 is established. The actuator pushes the aluminum cantilever beam when the voltage is applied. The maximum output torque of actuator can be obtained by calculating the deflection of the cantilever beam measured by the laser sensor. For the stick–slip piezoelectric actuator, the output force is closely related to the preload. Figure 19 shows the maximum output torque under different preloads when voltage is 300 V. In the range of 0.11 N to 1.01 N, the output torque increases with the increase in the preload and achieves the maximum value of 0.18 Nmm under a preload of 1.01 N. When the pretightening force exceeds 1.01 N, the output torque decreases continuously because the output force of the shear piezoelectric stack is gradually unable to overcome the larger friction.

### 4.4. Comparison of the Rotary Piezoelectric actuators

Table 1 compares the present actuators with those in the literature [15,31,35,36]. The concerned itemsmainly include resolution, maximumsize, drive voltage, andmaximumoutput speed. According to Table 1, although the piezoelectric actuator proposed in this paper requires a higher driving voltage, it shows some obvious advantages. The actuator not only has a small size, but also has a high resolution, and the output speed is relatively high.

## 5. Conclusions

This paper presents the design of a micro-rotating piezoelectric actuator based on a shearing piezoelectric stack. The structure of the actuator is compact, the overall size is 12 mm × 10 mm × 8 mm, and the mass is only 4.12 g. The bidirectional and large angle driving can be achieved using one shear piezoelectric stack. To further study the stepping characteristics of the actuator, a prototype was fabricated, and a series of experiments were conducted.

The data show that the maximum step size of the actuator can reach 547.8 μrad when the driving voltage is 300 V and the preload is 0.11 N. The maximum output speed is sufficiently high at 5.86 × 10${}^{5}$ μrad/s. The resolution is 0.64 μrad for a preload force of 1.01 N and a voltage of 30 V, and the maximum output torque is 0.18 Nmm.

In general, the experiment showed that the piezoelectric actuator not only offers good performance in terms of motion speed but also in terms of motion resolution. The micro-actuator can be used to drive light and small objects, such as in micro-/nano-processing, and cell operation. This study has great significance for the design of micro- and high-precision piezoelectric actuators.

## Figures and Tables

**Table 1:** Comparison of the rotary piezoelectric actuators.

Authors	Year	Resolution	Maximum Dimension	Drive Voltage	Velocity
This paper	N/A	0.64 μrad	12 mm	30–300 V	0.5 rad/s
Cheng G et al. [31]	2015	0.85 μrad	Over 90 mm	20–100 V	4.02 rad/s
Li J et al. [35]	2015	1.54 μrad	Over 20 mm	35–100 V	0.032 rad/s
Wang S et al. [36]	2017	0.75 μrad	140 mm	9 V–120 V	N/A
Yingxiang L et al. [15]	2018	0.095 μrad	Over 160 mm	25–210 V	N/A
